# Prostate cancer stem cells: deciphering the origins and pathways involved in prostate tumorigenesis and aggression

**DOI:** 10.18632/oncotarget.2953

**Published:** 2014-12-10

**Authors:** Adrian P. Rybak, Robert G. Bristow, Anil Kapoor

**Affiliations:** ^1^ McMaster Institute of Urology, Division of Urology, Department of Surgery, McMaster University, ON, Canada; ^2^ St. Joseph's Hospital, Hamilton, ON, Canada; ^3^ Princess Margaret Cancer Centre (University Health Network), ON, Canada; ^4^ Departments of Radiation Oncology and Medical Biophysics, University of Toronto, Toronto, ON, Canada

**Keywords:** Prostate cancer stem cell, Self-renewal, Castration-resistant prostate cancer, MEK-ERK (MAPK) and STAT3, PTEN and PI3K/AKT

## Abstract

The cells of the prostate gland are dependent on cell signaling pathways to regulate their growth, maintenance and function. However, perturbations in key signaling pathways, resulting in neoplastic transformation of cells in the prostate epithelium, are likely to generate subtypes of prostate cancer which may subsequently require different treatment regimes. Accumulating evidence supports multiple sources of stem cells in the prostate epithelium with distinct cellular origins for prostate tumorigenesis documented in animal models, while human prostate cancer stem-like cells (PCSCs) are typically enriched by cell culture, surface marker expression and functional activity assays. As future therapies will require a deeper understanding of its cellular origins as well as the pathways that drive PCSC maintenance and tumorigenesis, we review the molecular and functional evidence supporting dysregulation of PI3K/AKT, RAS/MAPK and STAT3 signaling in PCSCs, the development of castration resistance, and as a novel treatment approach for individual men with prostate cancer.

## INTRODUCTION

Prostate cancer (PCa) is the most common non-cutaneous male malignancy and the second leading cause of cancer-related deaths in males, with an estimated 233,000 new cases (27% of all new cancer cases in males) expected to occur in the United States in 2014 [1]. During prostate tumorigenesis, oncogenic signaling pathways promote the progression of androgen-dependent carcinomas to castration-resistant (hormone-refractory) prostate cancer (CRPC), the major contributing factor in PCa fatalities. The primary treatment for localized PCa is radiotherapy (external beam or brachytherapy) or complete removal of the prostate (radical prostatectomy). However, patients relapse in 30-50% of intermediate to high risk cases and neoplastic growth of aberrant prostate cells resumes either locally or systematically [2]. Relapsed patients are often subjected to androgen deprivation therapy (ADT) in which either chemical or surgical castration is performed along with anti-androgen treatment. Tumor cell proliferation despite the use of androgen deprivation (e.g. increased prostate specific antigen (PSA) levels in serum regardless of ongoing treatment), defines the presence of CRPC [3].

Histological studies of PCa tissues have identified a specific lesion of prostatic intraductal dysplasia [4], now commonly referred to as prostatic intraepithelial neoplasia (PIN). PIN is characterized by the appearance of luminal epithelial hyperplasia, enlarged nuclei and nucleoli, a reduction in basal cells while maintaining an intact basement membrane, and is generally regarded as a pre-neoplastic lesion in humans. The majority of prostate cancers are pathologically classified as adenocarcinoma and display a luminal phenotype, with the absence of staining for basal cell markers p63 and cytokeratins 5 and 14 (CK5/14) [5]. Less common histological variants of PCa include, among others, squamous cell carcinoma and small cell carcinoma [6]. While individual prognosis is related to the stage and histological grade of the index lesion (i.e. largest cancer in the prostate), PCa is multifocal in nature with focal lesions having been found to possess different genetic abnormalities from one another [7]. The presence of multiple neoplastic lesions with unique genetic compositions in PCa suggests that different cells within prostate epithelia may be vulnerable and contribute towards tumorigenesis.

In this review, we define prostate stem cells (PSCs) as the cells capable of differentiating into single (unipotent) or multiple cell types (multipotent) that form the prostate epithelium. The PCa cell-of-origin refers to the cancer-initiating cell(s) within the prostate epithelium, whether it is a stem/progenitor cell itself or its differentiated progeny which acquire mutations that re-instigate self-renewal ability and prevent post-mitotic differentiation, resulting in the emergence of cancer stem cells [8]. Prostate cancer stem cells (PCSCs) are a subset of cells within the prostate tumor that display tumor-propagating ability, demonstrate long-term self-renewal potential, and support a model in which survival from radiotherapy, chemotherapy and development of castration-resistant disease is met with an increased proportion and/or activity of PCSCs. Therefore, understanding the cell-of-origin and the role of PCSCs in prostate tumorigenesis, the signaling pathways that promote PCSC maintenance as well as their involvement in tumor progression will be necessary towards achieving novel therapeutic approaches towards combating PCa.

### CELLULAR HIERARCHY IN NORMAL MURINE AND HUMAN PROSTATES

**Figure 1:**
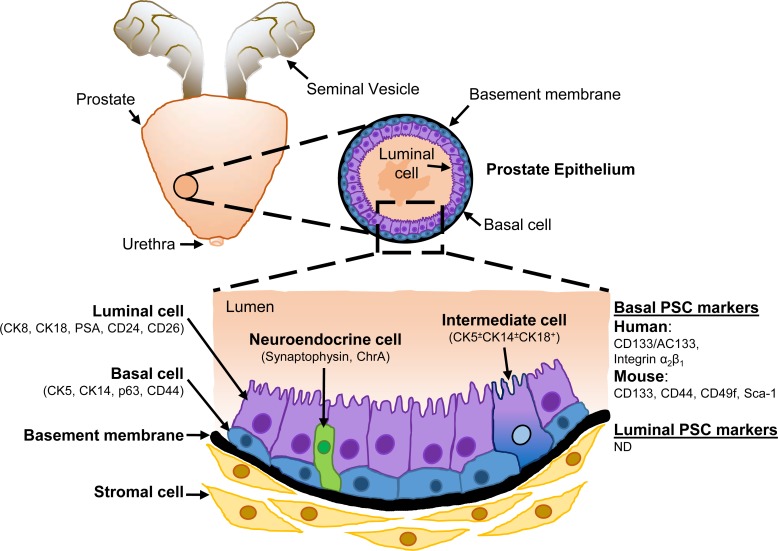
Schematic representation of the cellular architecture of the prostate epithelium The prostate epithelium consists of an inner layer of secretory luminal cells. Basal cells form a continuous layer of cells around the luminal cells and in contact with the basement membrane, which serves as a barrier between the epithelium and stromal compartment. Intermediate cells display both basal and luminal markers, while rare neuroendocrine cells are scattered throughout the epithelium. Markers used to distinguish each cell type are indicated in brackets, along with the surface markers used to identify human and murine prostate stem cells (PSCs). *ChrA* Chromogranin A; *ND* not determined.

The prostate contains epithelial cells of various lineage hierarchy that compose this glandular tissue. In adult males, the prostate gland contributes to the production of secretory proteins which are present within the seminal fluid [5]. Within the prostate epithelium, three epithelial cell types exist: luminal secretory cells, basal cells and intermediate cells. Luminal cells are a differentiated androgen-dependent cell type that produce and release prostatic secretory proteins into the lumen including PSA [9], a serine (Ser) protease responsible for preventing semen coagulation during ejaculation [10]. Luminal cells are characterized by the expression of androgen receptor (AR) [11], CD24 and CD26 cell surface proteins [12], as well as CK8 and CK18 [13]. Basal cells, which are located between luminal cells and the basement membrane, express CD44, p63 and CK5/14 proteins [13-15], display low AR protein levels [11] and lack PSA expression [9]. Intermediate cells are luminally-located cells in budding acini during prostate development that express cytokeratins found in basal and luminal cells [16], and are associated with luminal differentiation of basal cells [16-18]. Dispersed among basal and luminal cells are neuroendocrine cells, a rare AR-negative cell type in the prostate epithelium [19] (Figure 1).

#### Prostate stem cells in murine tissues

Tissue-specific stem cells are defined by their capacity for long-term self-renewal and to produce mature progeny, which include non-renewing progenitors and terminally-differentiated cells that constitute distinct cell types within the tissue of interest [20]. Self-renewal is the ability of stem cells to maintain an undifferentiated state through cell division without losing their identity or functional potential, thus ensuring maintenance of the stem cell population during clonal growth [21-23]. The concept of a stem cell compartment in the prostate epithelium was first realized upon evaluating the regenerative capacity of the prostate following castration-induced atrophy in adult rats [24, 25]. Castration results in prostate regression in response to androgen deprivation, with a stable number of cells remaining in a regressed state. Upon re-administration of androgen, the prostate epithelium regenerated over a two-week period [24, 25]. The ability for the prostate to undergo several rounds of regression and regeneration following androgen ablation and restoration, respectively [26], indicates that the prostate contains a long-term surviving population of PSCs that are resistant to castration.

In the mouse prostate, there is evidence for distinct PSCs with either a basal or luminal phenotype. Prostate cells expressing stem cell antigen-1 (Sca-1) reconstitute secretory-producing prostatic ducts lined with basal and luminal cells, which form upon combining Sca-1^+^ cells with embryonic urogenital sinus mesenchyme (UGSM) cells under the renal capsule of mice [27]. Using specific cell surface markers to further discriminate prostate basal (CD49f^+^) Sca-1^+^ cells from luminal (CD24^+^CD49f^−^), stromal (CD34^+^), haematopoietic (CD45^+^, Ter119^+^), and endothelial (CD31^+^) cell lineages (Lin), purified Sca-1^+^CD49f^+^Lin^−^ cells demonstrated self-renewal ability *in vitro* and formed *in vivo* prostatic ducts containing basal and luminal cells [28]. Furthermore, a single murine prostate cell, defined by the Sca-1^+^CD133^+^CD44^+^CD117^+^Lin^−^ marker profile, generated a secretion-producing prostate when transplanted with UGSM cells under the kidney capsule [29].

Although the functional prostate regeneration assay has demonstrated that murine prostate basal cells are capable of being bipotent, generating both basal and luminal cell lineages, such tissue reconstitution assays involve co-culturing basal cells with UGSM cells [27-29] which provides a strong inductive influence on prostate cells during engraftment [30]. To avoid any unexpected plasticity that may manifest upon removing prostate cells from their endogenous tissue microenvironment, genetic lineage-tracing experiments have explored the nature of prostate basal or luminal cells towards forming the prostate epithelium *in situ* following castration-driven prostate regression and androgen-mediated prostate regeneration studies. Expression of a tamoxifen (TAM)-inducible Cre-recombinase (Cre) driven by the *CK5* promoter labelled rare basal cells within the prostate epithelium that produced both basal and luminal cell progeny *in situ* following androgen-mediated regeneration [26]. Similarly, basal cells in the developing and adult mouse prostate were observed to be multipotent, giving rise to basal, luminal and neuroendocrine cells following cell lineage analysis [17, 31], while prostate luminal progenitors contribute to luminal cell expansion during postnatal development [17]. These findings contrast with the results of recent reports indicating that prostate basal and luminal cell lineages are self-sustaining (unipotent) in the adult mouse prostate and do not typically undergo lineage conversion *in situ* [18, 32], with prostate basal cells requiring inflammatory cues to demonstrate plasticity and generate luminal cells [18].

Additional evidence supports the existence of PSCs that are of luminal cell origin. The *p63*-null prostate lacks basal cells, however, prostatic ducts containing luminal cells formed upon grafting *p63*-null prostate epithelial cells along with UGSM cells and supplementing with testosterone [33]. Furthermore, expression of a TAM-inducible Cre driven by the *PSA* promoter labelled prostate luminal cells that were capable of surviving castration and reconstituting the luminal cell compartment *in situ* following androgen treatment [34]. A population of castration-resistant Nkx3.1-expressing (CARN) cells, which display a luminal phenotype in the regressed prostate, generated prostate basal and luminal cells following androgen-mediated regeneration, indicating that CARN cells are bipotent in nature [35]. Therefore, regenerated prostate luminal cells appear to be derived from pre-existing luminal cells that survive castration [32, 34, 35]. The reason for these discrepancies is unclear at present and suggests that the prostate cell lineage hierarchy has not been clearly characterized, with distinct PSCs with different plasticities existing in the mouse prostate.

#### Prostate stem cells in human tissues

In the human prostate, initial evidence supported PSCs confined to the basal cell compartment. Human prostate cells with a basal phenotype undergo self-renewal *in vitro* [36], with the capacity to reconstitute the prostate epithelium containing basal and luminal cells in a prostate regeneration assay [36, 37]. The recent establishment of organoid cultures using human prostate epithelial cells isolated from primary tissues has demonstrated that both basal (CD49f^+^) and luminal (CD24^+^, CD26^+^) cell populations contain bipotent cells which retain the ability to differentiate towards prostate basal and luminal cell lineages *in vitro*, as well as in tissue reconstitution assays involving UGSM cells [38]. However, prostate organoids derived from human and mouse prostate epithelial cells do not generate prostate neuroendocrine cells [38, 39], suggesting that organoid culture conditions need to be optimized to promote neuroendocrine differentiation. Despite this incomplete reconstitution of the prostate epithelium by prostate epithelial cells in culture, organoids derived from prostate luminal cells more closely resemble prostate glands than those derived from prostate basal cells [38, 39], and therefore, they may be more physiologically relevant in studies which attempt to recapitulate *in situ* histology in an *in vitro* setting.

A number of cell surface markers have been used to identify prospective human PSCs. CD44, which binds the extracellular matrix component hyaluronate [40], enriched for human PSCs in the basal cell layer possessing prostate regenerative activity [36]. Rapid adhesion to type I collagen isolated a population of prostate epithelial cells expressing high cell surface levels of integrin α_2_β_1_ (α_2_β_1_^hi^), another prospective PSC marker. Primary prostate α_2_β_1_^hi^-expressing cells formed slow-proliferating colonies in culture displaying a basal phenotype (PSA^−^CK5/14^+^) and produced epithelia consisting of basal and secretory luminal (PSA^+^AR^+^) cells when combined with stromal cells and implanted in recipient mice [41], which was considered as evidence of PSC activity. CD133, a five-transmembrane domain cell surface glycoprotein [42], is another putative PSC marker. Monoclonal antibodies have been developed against distinct epitopes of CD133, the most commonly used being AC133 (CD133/1) and AC141 (CD133/2), with CD133/AC133 enriching for human haematopoietic and neural stem cells [43, 44]. CD133/AC133^+^ human prostate epithelial cells, which are restricted to the integrin α_2_β_1_-expressing basal cell subpopulation and account for less than 1% of prostate basal cells, generated prostate acini *in vivo* that resemble prostate epithelia consisting of basal and luminal cells [45] (Figure 1). Whether these cell surface markers highlight basal-derived PSCs in human prostate organoid cultures will likely be addressed in future studies, while the discovery of novel cell surface markers corresponding to PSCs of luminal cell origin, which at present are unknown, are enthusiastically anticipated.

### CELL-OF-ORIGIN IN PROSTATE CANCER

The PCa cell-of-origin refers to the cell(s) within the prostate epithelium, whether it be a PSC or its lineage-restricted progeny, which acquires tumor-promoting mutations and subsequently initiates tumorigenesis [8]. At present, there is insufficient evidence supporting prostate cells which display prospective PSC markers as serving as the cell-of-origin in human PCa. Taylor and colleagues reported that the CD133/AC133^+^ PSC population may not serve as the cellular target responsible for prostate tumor initiation [46]. CD133/AC133^+^ cells from SV40-immortalized prostate cells were observed to be incapable of generating tumors when recombined with UGSM cells or cancer-associated fibroblasts in immunocompromised mice, whereas CD133/AC133^−^ cells formed xenograft tumors [46]. While the CD133/AC133^+^ PSC population may acquire oncogenic mutations, its differentiated (CD133/AC133^−^) progeny may subsequently undergo oncogenic transformation and correspond to the PCa cell-of-origin. Similarly, genetic lineage-tracing in a glioma mouse model has shown that neural stem cells acquire oncogenic mutations, however, it is their descendent oligodendrocyte progenitor cells that act as tumor-initiating cells during gliomagenesis [47]. Therefore, an alternative approach towards investigating the cell-of-origin for PCa has involved studying the vulnerability of prostate basal and luminal epithelial cells towards tumor initiation using transgenic mouse models of PCa, with cell surface marker expression being utilized to characterize the tumor-propagating cell population which sustains prostate tumorigenesis.

#### Basal versus luminal cell origin for PCa

Elucidation of the cell lineage relationships within the prostate epithelium is relevant for understanding the origins of PCa. Given that prostate adenocarcinoma has a luminal phenotype, the cell-of-origin for PCa should correspond to a luminal cell that has undergone oncogenic transformation, or a basal cell that differentiates and proliferates as luminal progeny following tumorigenesis [5]. A basal cell origin for PCa has been suggested as human prostate basal cells expressing AR, AKT and ERG oncogenes formed carcinoma in tissue reconstitution assays that is histologically similar to clinical human PCa [37]. Human prostate luminal cells appear to be resistant to AKT and MYC-induced transformation, suggesting that additional oncogenes like eIF4E, a target of PI3K/AKT signaling, may be required to promote neoplastic transformation of prostate luminal cells [48].

Genetically-engineered animal models have been utilized to address the cell-of-origin in PCa. Murine prostate basal cells have been reported to undergo neoplastic transformation [26, 32, 49]. In contrast, deletion of the *Pten* (Phosphatase and tensin homolog deleted on chromosome 10) tumor suppressor gene in luminal CARN cells resulted in carcinoma formation following androgen treatment [35]. While prostate carcinomas generated following *Pten* deletion in prostate basal or luminal cells are histologically similar [26, 32], basal cells are relatively resistant towards oncogene-induced transformation *in situ* [32] with prostate basal cell-derived carcinoma arising slowly [26]. This long latency may be due to the loss of Pten activity inducing murine prostate basal cells to differentiate into transformation-competent luminal cells [32], which requires prostatic inflammation to accelerate this differentiation process [18].

Animal models also support a luminal cell origin for PCa. Loss of the *Nkx3.1* tumor suppressor gene, whose expression is restricted to luminal cells in human prostate glands [50], induced the formation of pre-neoplastic PIN-like lesions in murine prostates [50-52], with concurrent loss of *Pten* [53, 54] or overexpression of MYC oncoprotein [55] promoting the development of invasive carcinoma. PTEN ablation in prostate luminal cells is capable of driving PCa formation itself, as *PTEN* loss following *PSA* promoter-driven TAM-inducible Cre expression (*PSA^CreERT2^Pten^lox/lox^*) resulted in PIN formation (2-3 months) followed by invasive adenocarcinoma development (8-10 months) [56]. More recently, Shen and colleagues demonstrated through genetic lineage-tracing that prostate luminal cells, but not prostate basal cells, were consistently observed as the PCa cell-of-origin in a diverse range of mouse models (*Pten^+/−^*, *Nkx3.1^+/−^Pten^+/−^*, *TRAMP*, and transgenic *ARR_2_/probasin*-driven *Myc*) *in situ* [57]. Furthermore, luminal cell-derived murine PCa displays a molecular gene signature that is highly correlated with poor patient prognosis (reduced biochemical-free survival) and upregulated in high-risk patients [26].

In addition to influencing the tumor cell's malignant potential, recent evidence suggests that the nature of the cellular target within the prostate tumor may also influence tumor histopathology. Basal tumor cells can generate tumor lesions exhibiting a mixture of squamous and adenocarcinoma regions in tissue reconstitution assays, while luminal-like tumor cells generate tumors with solely an adenocarcinoma phenotype [48]. Therefore, cell-of-origin models propose that distinct cell types within the prostate cell lineage hierarchy are vulnerable towards tumorigenesis and may give rise to PCa subtypes with different histopathologies, prognoses and/or treatment responses. At least in a mouse model, luminal cell-derived prostate tumors seem to recapitulate the more aggressive types of human PCa [26], while *NKX3.1* haploinsufficiency, *PTEN* loss and/or allelic gain of the *c-MYC* gene in human PCa are associated with increased biochemical relapse (recurrence of PSA serum levels) following radiotherapy [58, 59]. This implies a scenario whereby human prostate tumors, which are associated with different prognoses and phenotypes, may originate from prostate basal and/or luminal cells, respectively.

### EVIDENCE OF PCSCs IN HUMAN PROSTATE CANCERS

PCa is characterized by the excessive and uncontrolled growth of abnormal cells that invade the basement membrane, metastasizing primarily to the lymph nodes or bone. Like many other carcinomas, PCa exhibits a functional hierarchy of cells with tumor-propagating cells positioned at the apex and having the ability to differentiate into a spectrum of less tumorigenic progeny [60, 61]. The cancer stem cell (CSC) hypothesis postulates that only a subset of cells within the tumor are capable of sustaining tumorigenesis and driving disease progression, while establishing the cellular heterogeneity that constitutes the primary tumor. Stem-like cancer cells, often referred to as CSCs or tumor-propagating cells, are defined by their ability to initiate tumors upon implanting in immunocompromised mice [62]. CSCs generate non-tumorigenic progeny, resulting in intrinsically different populations of tumorigenic and non-tumorigenic cells within the tumor [63]. Despite the limited experimental means to identify and evaluate CSCs *in situ*, the ability of an isolated, phenotypically distinct population of cancer cells to initiate tumor formation in immunocompromised mice is currently the standard assay available to define human CSCs *in vivo* [62].

Accumulating evidence supports the existence of human PCSCs. A small population of primary PCa cells expressing the cell surface antigenic profile CD133/AC133^+^CD44^+^α_2_β_1_^hi^ were suggested to be prospective candidates of PCSCs [64], as CSCs are known to share similar cell surface antigens with their normal tissue stem cell counterparts [65]. Consistent with this concept, human prostate epithelial cells and malignant cells express PSC markers CD44, CD133 and/or integrin α_2_β_1_ [66-68], and these prospective PCSCs generated xenograft tumors that resembled the original tumor [66]. These observations are supported by reports that CD44^+^ cells isolated from PCa cell lines display increased tumorigenicity *in vivo* compared to CD44^−^ cells [60], with the CD44^+^α_2_β_1_^+^ cell population being enriched in PCSCs [61]. Furthermore, PCa cells expressing CD133/AC141 and CD44 displayed an increased ability to form tumors *in vivo* compared to an equal number of CD133/AC141^−^CD44^−^ cells [69]. Despite these observations supporting the candidacy of CD133^+^CD44^+^α_2_β_1_^hi^ cells as prospective PCSCs, it still remains to be demonstrated whether CD133^+^CD44^+^α_2_β_1_^hi^ cells isolated from primary PCa tissues are capable of tumor initiation *in vivo*.

Due to the constraints in obtaining a sufficient amount of human PCa material to isolate and characterize prospective PCSCs, established PCa cell lines and/or xenograft models have become useful surrogate sources for investigating human PCSCs. Immortalized human prostate cells expressing integrin α_2_β_1_, but not CD133/AC133, formed xenografts with malignant characteristics [46], implicating proliferative CD133/AC133^−^ cells [45] as being more susceptible to tumorigenesis compared to CD133/AC133^+^ stem-like cells [64]. Human PCa cells derived from androgen-insensitive DU145 cells and propagated under serum-free conditions as non-adherent spheres express surface markers CD44, integrin α_2_β_1_ and CD24, both basal and luminal cytokeratins, and display increased tumorigenicity *in vivo* [70]. Using human primary PCa xenografts, CD133^+^ or CD24^+^ PCa cells, isolated from tumor growths and serially diluted, initiate secondary tumor xenograft formation at comparable frequencies [71]. Moreover, a small subset of cells isolated from the CWR22 orthotopic tumor xenograft model display TRA-1-60, CD166 and CD151 markers, with TRA-1-60^+^CD166^+^CD151^+^ cells initiating tumor formation *in vivo* at increased efficiency following serial dilution of cells compared to TRA-1-60^+^ cells [72]. Tumor xenografts derived from primary human PCa tissue also recapitulate histological features present in the original tumor, with castration resulting in tumor regression leaving residual stem-like cells which display tumor-regenerating potential following testosterone re-administration [73]. Therefore, PCa xenografts serve as an *in vivo* source of human PCa cells, which can be propagated following serial transplantation in a number of grafting sites and subsequently used to isolate prospective human PCSCs. However, the recent generation of patient-derived PCa organoid cultures that recapitulate the diverse mutational landscape and histology observed in their original PCa tissue samples [74] will likely further aid in the characterization of PCSCs, as well as improve the understanding of the molecular determinants of therapeutic and castration resistance.

### EVALUATION OF PCSC ACTIVITY

#### *In vitro* propagation and evaluation of self-renewal activity of PCSCs

Different culture methods have been utilized to propagate PCSCs derived from human PCa cell lines including suspension or low adherence culture [67, 68], and media cultures containing either fetal bovine serum [60, 61, 66] or serum-free media supplemented with epidermal growth factor [75, 76] and basic fibroblast growth factor [67, 69, 70, 72]. However, the formation of free-floating spheres under serum-free conditions is routinely used as an *in vitro* approach towards propagating and evaluating the stem-like properties of putative CSCs derived from solid tumors [62], including those derived from PCa cells [69, 70, 72]. Despite its limitations [77], the sphere formation assay allows for the clonality and self-renewal capacity of stem-like cells to be evaluated upon culturing individual cells, reducing the impact of progenitor-like cells which display limited self-renewal potential [78]. The self-renewal capacity of sphere-derived human PCSCs has been evaluated [70, 76], which display enhanced tumor formation *in vivo* [69, 70, 72, 76].

#### Functional identification of prostate cancer cells with stem-like properties

While purification of human PCa cells based on the expression of surface markers isolates stem-like cells with tumor-initiating ability (Table 1), flow cytometry can also enrich for prospective PCSCs based on their ability to efflux the Hoechst 33342 dye. These cells, also known as side population (SP) cells [79], isolated from androgen-sensitive LAPC9 cells are capable of *in vivo* tumor formation unlike the non-SP cell population [80]. The SP phenotype is associated with the expression of the ATP-binding cassette membrane transporter family of proteins, particularly ABCG2 [81], which are involved in drug efflux and confer multi-drug resistance [82]. However, ABCG2^+^ and ABCG2^−^ PCa cells displayed similar tumorigenic potential *in vivo* [80], implicating the expression of additional ABC transporter proteins and/or other means of Hoechst 33342 dye efflux by these PCa cells.

**Table 1:** Marker profiles of prospective human prostate cancer stem-like cells (PCSCs)

PCSC Marker Profile	Source of PCSCs	Evidence for PCSC Activity *in vivo*
CD133^+^ or CD24^+^	1° PCa xenograft (SC; TS, IC mice)	Purified cells generate 2° tumors at comparable frequencies (at limiting dilution) upon serial transplant [71].
CD133/AC141^+^CD44^+^	DU145 cells (monolayer and sphere culture)	Purified cells display increased tumor-initiating ability in male IC mice [69].
CD44^+^ integrin α_2_β_1_^+^	Human PCa cell lines (monolayer cell culture)	CD44^+^ PCa cells are more tumorigenic than CD44− cells; CD44^+^ integrin α_2_β_1_^+^ cell population is enriched in PCSCs [60, 61].
CD44^+^CD24^+^ integrin α_2_β_1_^+^	DU145 spheres	Sphere cells display increased tumor-initiating ability in male IC mice [70].
CD44^+^ALDH^hi^ integrin α_2_β_1_^+^	LAPC9 xenograft (SC; castrated male, IC mice)	10^1^ sorted PCa cells form 2° tumors in castrated IC mice compared to 10^4^ CD44-ALDH^low^ integrin α_2_β_1_^+^ - cells [91].
TRA-1-60^+^CD166^+^CD151^+^	CWR22 xenograft (OT; intact male, IC mice	Triple marker-positive PCa cells display enhanced tumor-initiating ability compared to TRA-1-60^+^ cells [72].
HLAI^−^	Human primary PCa cells and cell lines	HLAI^−^ PCa cells display increased tumorigenic potential in IC mice compared to HLAI^+^ cells [162].

Abbreviations: 1° primary; 2° secondary; *hi* high activity; *HLAI* human leukocyte antigen class I; *IC* immunocompromised; *OT* orthotopic; *PCa* prostate cancer; *TS* testosterone-supplemented; *SC* subcutaneous.

An alternative strategy towards purifying PCSCs involves measuring their aldehyde dehydrogenase (ALDH) activity. Elevated ALDH activity has been associated with CSCs in various solid tumors [83] and is required to maintain the drug-tolerant cancer cell subpopulation [84]. ALDH1A1, which is expressed by a minor basal cell population in human prostates [85], was observed to be heterogeneously expressed in prostate adenocarcinomas, with elevated ALDH1A1 expression being positively correlated with the severity (Gleason score and pathologic stage) of the disease and inversely correlated with patient survival [85, 86]. Primary and metastatic PCa also express ALDH7A1 protein [87], with knockdown of ALDH7A1 reducing the tumor-propagating and metastatic abilities of androgen-independent PCa cells [88]. Using a functional (Aldefluor™) assay validated to assess ALDH1 isoform activities [89, 90], PCa cells demonstrating high ALDH activity (ALDH^hi^) were isolated and displayed increased tumorigenicity *in vivo* [87]. In addition, as few as 10^1^ ALDH^hi^CD44^+^α_2_β_1_^+^ cells isolated from LAPC9 (low PSA-expressing; PSA^low^) tumor xenografts generated secondary tumors in castrated mice whereas 10^4^ ALDH^low^CD44^−^α_2_β_1_^−^ cells were required for tumor formation, indicating that the ALDH^hi^CD44^+^α_2_β_1_^+^ phenotype in a PSA^low^ cell population is enriched for castration-resistant PCSCs [91]. While 19 ALDH isoforms have been identified in humans [83], other ALDH isoforms have yet to be shown to be functionally-active in PCSCs or required for tumor formation *in vivo*. As no individual method isolates bona-fide human PCSCs exclusively, a combination of methods (sphere culture, and isolation based on cell surface marker expression and/or functional activity) can be used to enrich for and characterize human PCSCs, with subsequent implantation in immunocompromised mice to evaluate their tumor-propagating activity and serial transplantation to assess their long-term self-renewal capability *in vivo* [62].

### CELL SIGNALING PATHWAYS UTILIZED BY PROSTATE CANCER AND THEIR TUMOR-PROPAGATING CELLS

**Figure 2:**
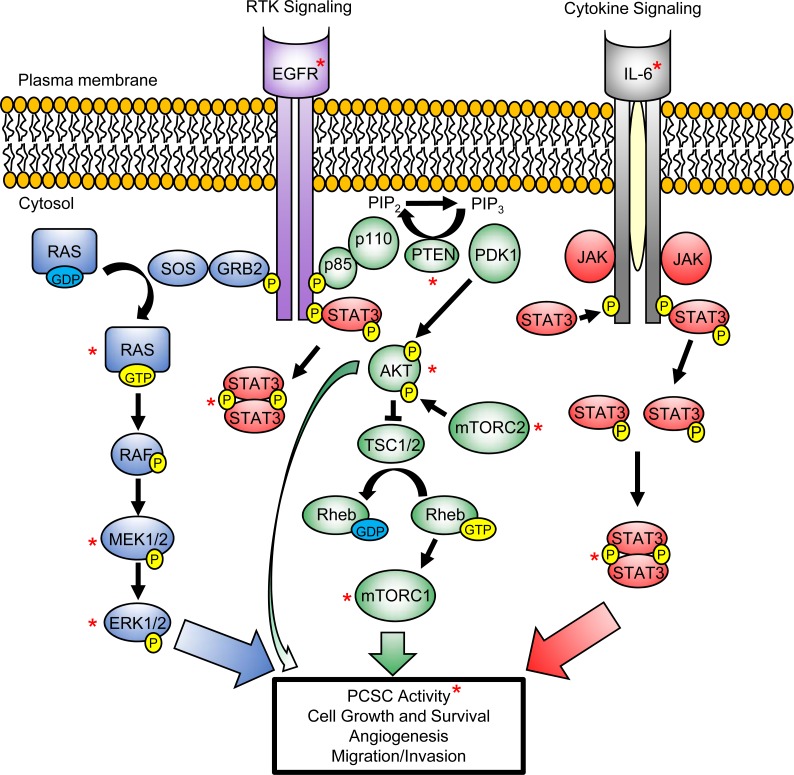
PI3K/AKT, RAS/MAPK and STAT3 signaling pathways converge to regulate PCSC maintenance and promote tumorigenesis Activation of PI3K/AKT (green), RAS/MAPK (blue) and STAT3 (red) signaling pathways, mediated by the activation of growth factor-driven receptor tyrosine kinase (RTK) (e.g. Epidermal growth factor receptor (EGFR)) or cytokine (e.g. IL-6) signaling, promote PCSC self-renewal activity and the various hallmarks of PCa development. These signaling pathways act directly, or through cross-talk activation, to mediate prostate tumorigenesis. *P* denotes phosphorylation of protein at specific residue(s), which is required for its activation (yellow). Red asterisk (*) marks key proteins within these signaling pathways that have been implicated in PCSC activity.

**Figure 3:**
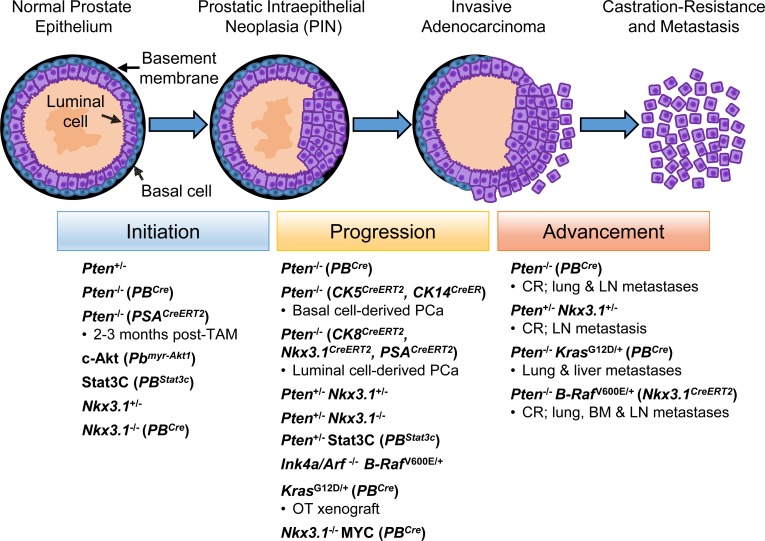
Prostate cancer initiation, progression and advancement are associated with proto-oncogene activation and inhibition of tumor suppressor genes involved in PI3K/AKT, RAS/MAPK and STAT3 signaling Abnormalities in the prostate epithelium result in pre-neoplastic lesions called prostatic intraepithelial neoplasia (PIN), which feature luminal epithelial hyperplasia and a reduction in the number of basal cells. PIN lesions progress to invasive adenocarcinoma (luminal phenotype) with loss of the basal cell layer and basement membrane resulting in various tumor grades, beginning with indolent to more aggressive forms of PCa, and subsequent development of metastasis and castration resistance. Studies involving murine PCa models provide support for the role of proteins involved in PI3K/AKT, RAS/MAPK and STAT3 signaling at various stages of PCa development. Murine PCa models discussed in the text are illustrated, with specific promoter-driven gene knockout or transgene overexpression indicated in brackets. Figure is adapted from ref. [5]. *BM* bone marrow; *c-Akt* constitutive-active Akt; *CR* castration-resistant; *Cre* Cre-recombinase; *CreER/CreERT2* TAM-inducible Cre; *LN* lymph node; *OT* orthotopic; *PB* probasin promoter; *TAM* tamoxifen.

A number of cell signaling pathways have been implicated in PCa progression towards an androgen-resistant state including receptor tyrosine kinases, like epidermal growth factor receptor (EGFR) [92-94], and developmental pathways including Wnt, Notch and Hedgehog signaling [95-97]. These developmental pathways have also been implicated in PCSC maintenance [98-100]. Human PSCs and PCSCs display low levels or lack AR expression [45, 64, 66, 72], with evidence to suggest that murine PSCs and PCSCs are capable of surviving in an androgen-independent state [26, 32, 101]. While not to undermine the importance of these developmental pathways and AR signaling in PCa, we will focus on the involvement of intracellular PI3K/AKT, RAS/MAPK and STAT3 pathways in maintaining PCSCs. Given that details of these signaling pathways and their ability to regulate various hallmarks of PCa have been reviewed thoroughly elsewhere [102-104], we highlight key proteins within these pathways that regulate PCSC activity (Figure 2), and the animal models that support their role in PCa development (Figure 3).

#### PI3K/AKT signaling

Dysregulation of PI3K/AKT signaling has been implicated in PCa given the discovery that its negative regulator, PTEN, is mutated and frequently lost in PCa. Loss of PTEN function, as a result of mutation, deletion or reduced expression, occurs at a frequency of ~40% in PCa [105, 106]. Integrative analysis of mRNA expression, copy number and exon sequencing for somatic mutations conducted on a cohort of PCa patients revealed that the PI3K/AKT pathway was altered in 42% of primary cancers and 100% of metastases, while the RAS/MAPK pathway was altered in 43% of primary specimens and 90% of metastases [106]. Both *PTEN* loss and AKT activation, as measured by Ser 473 phosphorylation, are associated with biochemical relapse following radical prostatectomy [107, 108], while *PTEN* deletion (along with allelic *c-MYC* gain) is associated with biochemical relapse following local therapy [59]. Intense staining patterns for activated AKT are correlated with higher Gleason grade PCa [109] and tumor progression [110], while *PTEN* loss is associated with PCa progression and predicts a shorter time for metastasis-free survival in patients [111].

The role of PI3K/AKT signaling in PCa has also been examined in animal models. *Pten*^+/−^ mice were first reported to display hyperplastic lesions in the prostate, with benign PIN lesions being observed in some mice at a young age (≤14 weeks) [112, 113]. *Pten* deletion in prostate basal or luminal cells has also been carried out to promote PI3K/AKT signal activation in order to determine the prostate cell lineage responsible for subsequent tumor initiation [26, 32, 35], as previously discussed. Prior to these studies, *probasin* (*PB*) promoter-driven *Pten* deletion was conducted, resulting in PI3K/AKT signal activation specifically in both prostate basal and luminal cells [114]. By 6 weeks of age, these mice formed PIN lesions, developed adenocarcinoma that invades stromal regions (9 weeks) and displays androgen-independent survival following castration, and generated metastases in the lymph nodes and lungs (12 weeks) [114]. Moreover, prostate epithelial cells lacking *Pten* require mammalian target of rapamycin (mTOR) complex 2 (mTORC2) for neoplastic transformation [115], thereby implicating downstream mTOR signaling in murine PCa development. Expression of a constitutively-active form of Akt1 (c-Akt) resulted in PIN formation when expression is driven by the *PB* promoter [116], or after reconstituting c-Akt-expressing prostate basal cells with UGSM cells and grafting under the kidney capsule of mice** [49]. Therefore, the murine prostate epithelium has been shown to be vulnerable to *Pten* loss, with the propensity to undergo AKT-driven tumorigenesis.

The role of PI3K/AKT signaling in the proliferation and maintenance of PCSCs has been examined in human PCa. PTEN knockdown in DU145 PCa cells resulted in an increased ability to enrich for CD133/AC141^+^CD44^+^ stem-like cells [69]. Treatment with PI3K inhibitor LY294002 reduced sphere formation [69] while the dual PI3K/mTOR inhibitor NVP-BEZ235 reduced the CD133/AC141^+^CD44^+^ cell population *in vivo* and subsequently delayed tumor formation [117]. However, DU145 spheres display low levels of AKT activation while PTEN knockdown marginally increased AKT activation and did not affect sphere maintenance, suggesting that PTEN function may not be critical for PCSC self-renewal *in vitro* [70]. While PTEN may not be required to negatively-regulate PI3K/AKT-dependent PCSC maintenance, whether PTEN loss/inactivity and subsequent PI3K/AKT signal activation promotes PCSC differentiation into non-tumorigenic bulk tumor cells has not been thoroughly evaluated, despite PTEN knockdown increasing tumor formation *in vivo* [69].

#### RAS/MAPK signaling

Mitogen-activated protein kinases (MAPKs) are cytoplasmic Ser/threonine kinases that transduce signals by activating extracellular signal-regulated kinases (ERKs) through the RAS-RAF-MEK-ERK (RAS/MAPK) signal transduction cascade [118]. Similar to PI3K/AKT signaling, MAPK signal activation correlates with disease progression, with elevated staining for phosphorylated (active) forms of ERK1 and ERK2 (ERK1/2) being detected with increasing tumor stage of human PCa specimens [119]. Increased ERK1/2 activation in patients receiving neoadjuvant hormone therapy, and in recurrent CRPC patients, has been observed [120]. Mutations of all three *RAS* isoforms (*KRAS*, *HRAS* and *NRAS*) have been detected in human PCa specimens (3 – 30%), particularly among Japanese men [121-124], while the activating mutant *B-RAF(V600E)* has been observed in ~10% of Korean PCa patients [122]. These correlative studies reinforce that activated RAS/MAPK signaling contributes towards prostate tumorigenesis.

Further evidence in support of a relationship between RAS/MAPK signaling and PCa progression towards an androgen-independent state has been demonstrated in the preclinical setting. Ectopic expression of HRAS(T35S) in androgen-sensitive LNCaP cells maintained MAPK signal activation under serum-free conditions and increased their *in vivo* tumorigenicity compared to parental cells. More than two-thirds of these HRAS-expressing PCa xenografts were resistant to castration and displayed robust activation of MAPK signaling post-castration [125]. Conversely, inducible expression of dominant-negative HRAS(S17N) in the androgen-insensitive, highly tumorigenic derivative LNCaP cell line, C4-2, inhibited MAPK signaling and restored androgen sensitivity to these cells, causing tumor regression in surgically-castrated mice [126]. Furthermore, ERK1/2 activation is necessary and sufficient to mediate RAF-induced AR downregulation in PCa cells [127], supporting MAPK signaling in PCa advancement.

As studies suggest that RAS-driven MAPK activation plays a substantial role in PCa progression towards CRPC development and metastasis, the role of MAPK signaling in prostate tumor formation is less defined. DU145 PCSCs require MAPK signaling for their propagation *in vitro*, as treatment with MEK inhibitor U0126, ectopic expression of dominant-negative MEK1(K97M), or knockdown of either ERK1 or ERK2 reduced their sphere-forming ability [75]. Murine PCSCs generated as a result of expressing constitutively-active Kras(G12D) (*PB^Cre^Kras^G12D/+^*) or deleting *Pten* (*PB^Cre^Pten^lox/lox^*) in prostate epithelial cells initiated orthotopic tumor formation, while neither PCSC population was capable of generating detectable macrometastases by 10 weeks post-transplantation. However, PCSCs demonstrating concurrent activation of both PI3K/AKT and RAS/MAPK signaling (*PB^Cre^Pten^lox/lox^Kras^G12D/+^*) promoted metastasis to the lymph nodes, lung and liver, with MEK inhibitor (PD325901) treatment reducing orthotopically-engrafted tumors and inhibiting their ability to metastasize [120]. Similarly, *PTEN* loss and B-Raf(V600E) expression following *Nkx3.1*-driven TAM-inducible Cre expression (*Nkx3.1^CreERT2^Pten^lox/lox^B-Raf^V600E/+^*) resulted in murine prostate tumors that were inherently castration-resistant and which metastasized to the lung, lymph nodes and bone marrow [128]. While these studies implicate concomitant activation of PI3K/AKT and RAS/MAPK signaling in PCa advancement, activation of MAPK signaling itself has been demonstrated to promote basal (p63^+^) cell proliferation and the development of invasive prostate adenocarcinoma independently of PI3K/AKT signal activation [129], supporting its involvement in prostate tumorigenesis.

#### STAT3 signaling

Signal transducer and activator of transcription (STAT) proteins have been implicated in prostate tumorigenesis, particularly STAT3 protein [130, 131]. Activated STAT3 (phosphorylation at tyrosine 705) is associated with higher Gleason score and pathological stage of the disease [132, 133]. STAT3 activation is also associated with decreased survival in PCa patients [133], particularly in CRPC patients [134], and a shorter time to death from biochemical relapse [133]. In patients having undergone radical prostatectomy or hormonal therapy, recurrence-free survival rates were lower in patients displaying increased levels of JAK1 and STAT3 activation [133]. Elevated serum levels of IL-6, a known activator of STAT3 signaling, were observed in CRPC and metastatic PCa patients compared to patients bearing benign or non-malignant forms [135-137], suggesting that activated STAT3 signaling correlates with the clinicopathologic features of PCa.

In preclinical studies, STAT3 signaling promotes PCa development. Treating mice bearing subcutaneously-engrafted TRAMP-C1 PCa cells (TC1) with flutamide, an AR antagonist, immediately following engraftment resulted in elevated STAT3 activation compared to vehicle-treated mice. Moreover, knockdown of STAT3 in TC1 cells reduced tumor growth [138]. Substitution of alanine 662 and asparagine 664 for cysteine residues in Stat3 promotes its dimerization and generates a constitutively-active transcription factor (Stat3C) [139], with *PB* promoter-driven Stat3C expression promoting the development of PIN lesions while invasive adenocarcinoma developed when combined with *Pten* heterozygosity [140]. In human PCa cells, androgen-insensitive DU145 and PC3 cells display elevated STAT3 activation compared to androgen-sensitive LNCaP cells [141], with inhibition of STAT3 expression or activity inducing apoptosis of DU145 cells [141, 142]. Conversely, expression of constitutively-active Stat3C in LNCaP cells promoted androgen-independent tumor growth in castrated mice [143].

While STAT3 activation has been implicated in PCa development, its role in PCSCs has only recently been investigated. Pre-treating human PCSCs (CD133/AC133^+^α_2_β_1_^hi^) with increasing concentrations of STAT3 inhibitor LLL12 abrogated their tumor-propagating ability *in vivo* [144]. Spheres derived from human PCa cells display elevated STAT3 activation [75, 145] with STAT3 knockdown reducing sphere formation, while treatment with a soluble IL-6 receptor fusion protein inhibited STAT3 activation and prevented tumor growth *in vivo* [138]. Whether activated by cytokines such as IL-6 [144], stressors like reactive oxygen species [145] or driven by the progression towards castration resistance [138], STAT3 activation plays a vital role in the self-renewal and tumor-propagating capacity of PCSCs.

#### Cell signaling pathways: maintenance of the PCSC pool versus drivers of PCa development

Prostate tumorigenesis is a complex process which involves many hallmarks that are important beyond simply maintaining PCSCs within the tumor. Many factors are necessary for PCSC generation *in vivo*, and it can be envisaged that secondary events govern the growth and sustenance of the subsequent tumor mass. While it is intriguing that PCSCs utilize PI3K/AKT, RAS/MAPK and STAT3 signaling to self-renew and maintain their presence within the tumor, these same pathways are also required for PCa cell proliferation and survival, tumor angiogenesis and metastasis (Figure 2). Whether these signaling pathways play a critical role in forming and/or sustaining the prostate tumor, drive PCSC maintenance or simply are required for generating the bulk of the less tumorigenic cells (non-PCSCs) within the tumor will need to be addressed further in future studies. Recently, STAT3 signal blockade has been shown to inhibit the prostate tumor-propagating and bulk tumor cell populations in patient-derived PCa xenograft models, as well as block tumor angiogenesis [146]. Moreover, IL-6 driven STAT3 signaling induced the formation of a cancer stem-like population from non-stem PCa cells [146, 147], which could be inhibited by treatment with the small molecule STAT3 inhibitor, Stattic [146]. As CSC-like cells can arise *de novo* from non-tumorigenic cells [148] or following induction as a result of IL-6 secretion by CSCs [147], a dynamic equilibrium between CSCs and non-CSCs is suggested to exist in order to maintain a constant proportion of tumor-propagating cells over successive generations [147, 148]. The extent to which PI3K/AKT and RAS/MAPK signaling contribute towards this dynamic equilibrium along with STAT3 signaling in PCa, driven by IL-6 or perhaps other molecules, remains to be elucidated. Taken together, activated signaling appears to be required by both PCSC and non-PCSCs in order to drive and sustain prostate tumorigenesis through its various hallmarks, and is likely to be relevant in heterogeneous prostate cancers that maintain a balanced, yet small, number of PCSCs within a larger population of non-tumorigenic PCa cells.

### LESSONS FOR PROSTATE CANCER THERAPY: INHIBITION OF SIGNALING PATHWAYS AS A STRATEGY TO TARGET PCSCs

Active surveillance with serial PSA monitoring and prostate biopsies is a reasonable management option for patients with indolent, low-grade (≤Gleason 6) PCa [149]. However, 30% of patients diagnosed with low-grade PCa will transition to non-indolent cancers suggesting the presence of non-detected, aggressive tumor cell variants at the time of diagnosis [149] that are not reflected solely by Gleason score. This leads to the hypothesis that a higher proportion and/or activity of PCSCs may be present within these transitioning tumors. Higher grade PCa present as multifocal tumors [150] with genetic heterogeneity among foci [7], which suggests of multiple neoplastic transformation events occurring within the prostate and an increased number of PCSCs that may (or not) be genetically-distinct.

The standard treatment for non-metastatic, localized (intermediate and high risk) PCa remains to be external beam radiotherapy or radical prostatectomy, with strong evidence indicating that the efficacy of these therapies is related to the extent of killing local, proliferating tumor clones with PCSC properties [151-153]. Increased radiotherapy dose leads to increased clonogenic killing and the use of molecularly-targeted agents may lead to a better outcome by sterilizing radioresistant PCSCs [154, 155], providing improved local control and preventing the dissemination of resistant, proliferating stem-like PCa cells [156, 157]. A number of patients will also undergo biochemical relapse following localized therapy leading to the use of ADT, with progression-free survival lasting only several months following ADT compared to control treatment [158, 159] due to CRPC development. Similarly, docetaxel, an anti-mitotic chemotherapeutic agent, which is routinely used in treating patients whose disease progresses towards metastatic CRPC, provides a modest improvement in patient survival [160, 161]. Docetaxel-resistant PCa cells possess an increased number of cells with an undifferentiated phenotype due to the lack of low molecular weight cytokeratins (CK18 and CK19) and human leukocyte antigen (HLA) class I proteins, display elevated levels of AKT activation and pro-survival Bcl-2 protein expression, and demonstrate a higher tumor-propagating capacity *in vivo* compared to their parental, docetaxel-sensitive cells [162]. Therefore, along with enhanced radioresistance and chemoresistance [154, 162], development of castration-resistant disease serves as an adaptive or selective pressure for increasing the number and/or activity of PCSCs.

**Figure 4:**
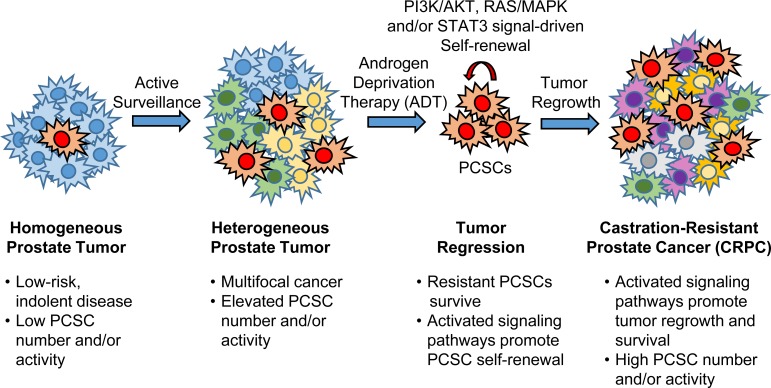
Reduced dependence on AR signaling promotes activation and subsequent cross-talk between PI3K/AKT, RAS/MAPK and STAT3 pathways to regulate PCSC maintenance and tumorigenesis Low-risk (indolent) PCa progresses during active surveillance to a heterogeneous tumor that is multifocal in nature. Conventional androgen-deprivation therapy (ADT) causes tumor regression by targeting androgen-responsive PCa cells. Inhibition of androgen receptor (AR) signaling promotes activation of cell survival pathways like PI3K/AKT, RAS/MAPK and STAT3, either directly or through cross-talk, in surviving androgen-independent PCSCs. Surviving PCSCs drive tumor regrowth and the development of castration-resistant prostate cancer (CRPC), with cell signal activation being sustained in castration-resistant disease.

The progression towards an androgen-resistant state is associated with elevated PI3K/AKT, RAS/MAPK and STAT3 signaling, signaling pathways that promote PCSC self-renewal and tumorigenesis, as previously described. Indeed, a reciprocal feedback loop exists between PI3K/AKT and AR signaling which regulates castration-resistant PCa growth [163, 164]. Neoplastic transformation of the prostate by cell autonomous PI3K/AKT signal activation occurs in the absence of epithelial AR expression or following androgen withdrawal [164], suggesting that androgen-targeted therapy may not be able to ablate PCSCs that display activated PI3K/AKT signaling and survive in an androgen-independent state. AKT also promotes phosphorylation of B-RAF (Ser 445) and its subsequent activation, with ERK1/2 activation requiring androgen deprivation in androgen-sensitive PCa cells [165]. Phosphorylation of Ser 727 residue on STAT3, which enhances its transcriptional activity [166], occurs in an ERK-dependent fashion [167], while ERK1/2 can activate mTOR complex 1 (mTORC1) directly [168] or through functional inactivation of tuberous sclerosis factor 2 (TSC2) [169] in order to promote RAS-dependent mTOR signaling. Furthermore, downregulation of AR signaling itself has been shown to promote a stem-like phenotype and increase the tumorigenicity of PCa cells through a STAT3 signal-dependent mechanism [138]. Taken together, a model can be proposed whereby reduced dependence on AR signaling promotes activation and subsequent cross-talk between PI3K/AKT, RAS/MAPK and STAT3 pathways to regulate PCSC maintenance and tumorigenesis, with sustained activation of these pathways in CRPC (Figure 4).

A combinatorial approach towards blocking multiple signaling pathways that are coordinately dysregulated in PCa has shown to be an effective strategy towards inhibiting PCa growth in the preclinical setting. Targeting PI3K/AKT/mTOR and MAPK pathways by combined PD325901 (MEK inhibitor) and rapamycin (mTOR inhibitor) treatment reduced prostate tumor burden of *Nkx3.1*^+/−^*Pten*^+/−^ mice, particularly following castration [110]. Furthermore, the combination of PD325901 and rapamycin treatment of mice bearing orthotopically-transplanted PCSCs displaying conditional *Pten* loss and oncogenic Kras(G12D) expression showed reduced primary tumor burden compared to PD325901 treatment alone, and ablated their ability for macrometastatic colonization [120]. This combined treatment was also effective in reducing the number of metastatic cases by one-third in mice displaying conditional *Pten* loss combined with oncogenic B-Raf(V600E) expression [128]. Finally, NVP-BEZ235 (PI3K/mTOR inhibitor) treatment in combination with docetaxel was more effective than chemotherapy alone at decreasing the human PCSC population and preventing tumor formation *in vivo* [117]. Taken together, these preclinical studies provide lessons and promise for treating advanced stage PCa (including metastatic CRPC) with the hope that a combinatorial strategy for PI3K/AKT/mTOR, RAS/MAPK and/or STAT3 signal inhibition will impose a series of blockades that will impact the tumor-propagating ability and maintenance of PCSCs, thereby lowering PCSC burden by altering the dynamic balance, and subsequent conversion, between non-PCSC and PCSC pools. Whether a pathway-targeted treatment regime, alone or in combination with current local, ADT, immunological or chemotherapeutic regimens, would lead to cure for individual patients at risk of systemic spread remains an area for future investigation. Therefore, pre-screening and subsequent monitoring of patient tumors for activated signaling and response to specific therapy will be required to determine the efficacy of such combinatorial therapy as a first-line or secondary treatment within the context of precision cancer medicine.

## CONCLUDING REMARKS

Despite PCa being a prevalent malignancy in men, our understanding of prostate tumorigenesis remains limited, including knowledge of its origin, as well as the factors and signaling pathways that regulate its initiation. Based on the reported tumor-propagating abilities demonstrated for CSCs in various solid tumors, PCSCs are believed to sustain prostate tumorigenesis and efforts have been made using cell-based and animal models to address their existence. Evidence in support of the existence of PSCs has been demonstrated in prostate regeneration experiments following castration and re-administration of androgen, with candidate PSCs displaying either basal or luminal cell characteristics in prostate glands that survive androgen depletion. Although aberrant cytokine-driven and receptor tyrosine kinase-driven signaling in prostate cells promotes PCa development, there is insufficient evidence demonstrating that PSCs acquire tumor-promoting mutations and serve as the PCa cell-of-origin, or whether it is their differentiated progeny that initiate prostate tumorigenesis. While PCa is generally accepted to be heterogeneous in nature, distinct subtypes formed due to the activation of different oncogenic pathways remain undefined, and whether human prostate basal and luminal epithelial cells are equally vulnerable to oncogenic transformation and subsequently account for the multifocality of neoplastic lesions within a given section of PCa tissue remains unclear. In addition, PCSCs are believed to be dynamic entities that co-exist in equilibrium with non-PCSC populations, which are likely to display variable phenotypes depending on their cell-of-origin and produce different PCa subtypes. A deeper understanding of PCa heterogeneity and multifocality, patient tumor profiling to determine the most effective combinatorial treatment, and subsequent monitoring of their tumors during treatment will be critical in achieving an efficacious response in personalized therapy.
